# Sudden Onset of Severe Pulmonary Hypertension in a Preterm Infant: A Case Report on the Role of Maternal Use of Serotonin Re-Uptake Inhibitors During Pregnancy and Concurrent Risk Factors

**DOI:** 10.3389/fped.2022.855419

**Published:** 2022-06-10

**Authors:** Isabella Buffoni, Silvia Buratti, Marisa F. Mallamaci, Stefano Pezzato, Elisabetta Lampugnani, Francesca Buffelli, Ezio Fulcheri, Andrea Moscatelli

**Affiliations:** ^1^Division of Neonatal and Pediatric Intensive Care, Emergency Department, IRCCS Giannina Gaslini Institute, Genoa, Italy; ^2^Fetal and Perinatal Pathology Unit, IRCCS Giannina Gaslini Institute, Genoa, Italy; ^3^Department of Surgical Sciences and Integrated Diagnostics, University of Genoa, Genoa, Italy

**Keywords:** pulmonary hypertension, preterm, newborn, selective serotonin reuptake inhibitors, case report, fetal growth restriction

## Abstract

Persistent pulmonary hypertension of the newborn (PPHN) is a severe condition caused by failed circulatory adaptation at birth. Pulmonary hypertension is most common in full-term infants and rare in preterms, although it is increasingly diagnosed also in extremely preterm infants. Previous studies demonstrated the association between maternal use of selective serotonin re-uptake inhibitors during gestation and pulmonary hypertension. This brief report describes the complex physiopathological correlations that were identified in a case of severe pulmonary hypertension in a fetal growth restricted (FGR) preterm infant, with a history of maternal use of antidepressants during pregnancy. Perinatal factors, triggers and aggravating mechanisms caused a dramatic clinical course. Maternal history of escitalopram therapy throughout pregnancy was noted. Uteroplacental insufficiency, fetal hypoxia, FGR, preeclampsia, preterm delivery, antenatal steroids, and cesarean section were documented as concurrent risk factors. Myocardial immaturity and dysfunction, secondary to FGR and prematurity aggravated the hemodynamic compromise. The short time gap between pharmacological ductal closure and the onset of PPHN may suggest a cause–effect relationship, as observed in previous reports. Placental histopathologic findings are reported.

## Introduction

Persistent pulmonary hypertension of the newborn (PPHN) is caused by disruption of the normal transition from fetal to neonatal circulation and it is associated with increased morbidity and mortality. PPHN occurs more frequently in full-term or near-term infants, with 1–2 cases per 1,000 live births (1, 2). Severe fetal asphyxia and meconium aspiration syndrome are the main risk factors, but several antenatal and perinatal conditions have been suggested to increase the risk for PPHN, including fetal growth restriction (FGR) and maternal use of selective serotonin re-uptake inhibitors (SSRIs) during pregnancy. Major depression affects up to 10% of pregnant women and SSRIs are the most common antidepressants used for medical treatment (3).

In preterm newborns pulmonary hypertension (PH) is increasingly recognized in the perinatal period and results from severe hypoxic respiratory failure soon after birth or significant pulmonary hypoplasia secondary to preterm premature rupture of membranes (pPROM) and oligohydramnios (4). In preterms pulmonary hypertension may present as a complication of bronchopulmonary dysplasia (BPD) (5).

PPHN is characterized by severe respiratory failure with profound hypoxemia, poorly responsive to supplemental oxygen secondary to extrapulmonary shunting. A difference in arterial partial pressure of oxygen (PaO2) ≥20 mmHg (2.6 kPa) or oxygen saturation ≥5–10% in simultaneous preductal and postductal measurements should be considered suggestive of PPHN. Echocardiographic evaluation documents right-to-left or bidirectional shunting at the foramen ovale (FO) and/or the ductus arteriosus (DA), altered shape of the interventricular septum (flattened or bowed to the left), as well as high pulmonary arterial/right ventricular systolic pressure estimated by Doppler velocity measurement of tricuspid regurgitation jet (2, 6).

The main therapy of PPHN includes treatment of the underlying causes, as well as supportive measures aimed to maintain cardiac output and optimize lung function. In this context, pharmacologic therapies such as inhaled nitric oxide (iNO), phosphodiesterase inhibitors (sildenafil), and synthetic prostacyclin analogs (epoprostenol) are routinely used (alone or in combination) to manipulate pulmonary vascular resistance (PVR) (7).

We report the case of a FGR 28-week-preterm infant with maternal history of escitalopram use during pregnancy who suddenly developed severe PPHN after pharmacological ductal closure. Antenatal and perinatal risk factors of PPHN have been identified after case analysis and correlated with PPHN physiopathology.

## Case Presentation

A preterm female infant was born at 28 weeks of gestational age (GA) with a birth weight of 660 grams (4th percentile) in our tertiary care center. Maternal history included a diagnosis of major depressive disorder, treated with escitalopram at a daily dose of 10 mg, from conception to the week before delivery. FGR was demonstrated early in pregnancy. Antenatal steroids were given. No pPROM was reported. The infant was delivered by urgent cesarean section due to preeclampsia and severe FGR with evidence of absent blood flow in the ductus venosus. The Apgar score was 6 at 1 min. She was intubated in the delivery room and transferred to the neonatal intensive care unit (NICU). In her first day of life (DOL), the infant was assisted with conventional pressure support ventilation at low parameters (fiO2 0.3), with a mild respiratory distress syndrome (RDS) pattern at chest X-ray and normal blood gas exchange. The first echocardiogram showed a hemodynamically significant DA with a size of 2.5 mm, bidirectional flow pattern and a left atrium:aorta (LA:Ao) ratio of 2 (severe left atrium dilation is defined as LA:Ao ≥ 1.5) (8); no abnormalities in the aortic arch anatomy were reported. On DOL 2 echocardiographic findings were unchanged and intravenous paracetamol treatment was started at a dosage of 15 mg/kg/dose every 6 h (Paracetamol, B. Braun, Melsungen, Germany) in 30 min, according to the “early targeted” strategy for patent DA treatment and considering the high risk of patent DA complications and low probability of spontaneous closure (8, 9). On DOL 3, the newborn developed multiple episodes of profound desaturation (SpO2 60–70%), increased pre- and post-ductal saturation difference (>20%), systemic hypotension (mean arterial pressure 21–23 mmHg) and tachycardia (heart rate 200 bpm). The newborn was unresponsive in absence of sedation, with distended abdomen (X-ray negative for pneumoperitoneum and other signs of necrotizing enterocolitis) and with mottled skin and cold extremities. Rule out sepsis work-up was completed and empiric antibiotic therapy was started. Clinical deterioration progressed rapidly with hemodynamic failure, oliguria and severe metabolic acidosis [pH 6.9, base deficit −16, lactate 60 mg/dl (6 mmol/L)]. The echocardiogram showed elevated right ventricle (RV) pressures, moderate RV dilation with systolic dysfunction and closure of DA. The patient was sedated, paralyzed and iNO was administered up to 20 parts per million (ppm). High frequency oscillatory ventilation support was implemented with fraction of inspired oxygen (FiO2) increased to 1.0. Despite volume resuscitation (normal saline 10 ml/Kg), high dose epinephrine (up to 0.2 micrograms/kg/min) and hydrocortisone (1 mg/kg/dose Q6 h) hypotension persisted, and furosemide continuous infusion was started as the patient developed anuria and anasarca. On maximal support the echocardiographic signs of severe PH did not improve confirming suprasystemic pulmonary pressures, D-shaped interventricular septum, RV dysfunction and closure of DA, therefore a second-line treatment with epoprostenol infusion at 15 nanograms/kg/min was added on DOL 5. Progressive improvement of PPHN (subsystemic pulmonary pressures, normalized interventricular septum morphology and RV function at echocardiogram assessment) and end-organ perfusion, demonstrated by increased urine output and resolution of metabolic acidosis, allowed rapid de-escalation of hemodynamic and ventilatory supports. Nasogastric sildenafil was started on DOL 7. Epoprostenol infusion and iNO were gradually weaned off by DOL 9. The patient was extubated on DOL 10 and definitively weaned off non-invasive ventilation on DOL 55. Sildenafil was gradually reduced and discontinued before discharge. Repeat echocardiogram assessment at 3 months of life showed complete resolution of PH and RV dilatation. The brain ultrasound monitoring was repeatedly negative for neurological complications. The infant has been enrolled in our follow up program for high-risk infants after discharge, that occurred on DOL 92. The patient is currently well with no evidence of PH recurrence and a normal echocardiogram 24 months after birth. Neurological development is adequate with normal brain magnetic resonance. The growth curve is satisfactory despite difficulties in oral feeding during weaning.

**Figure 1:**
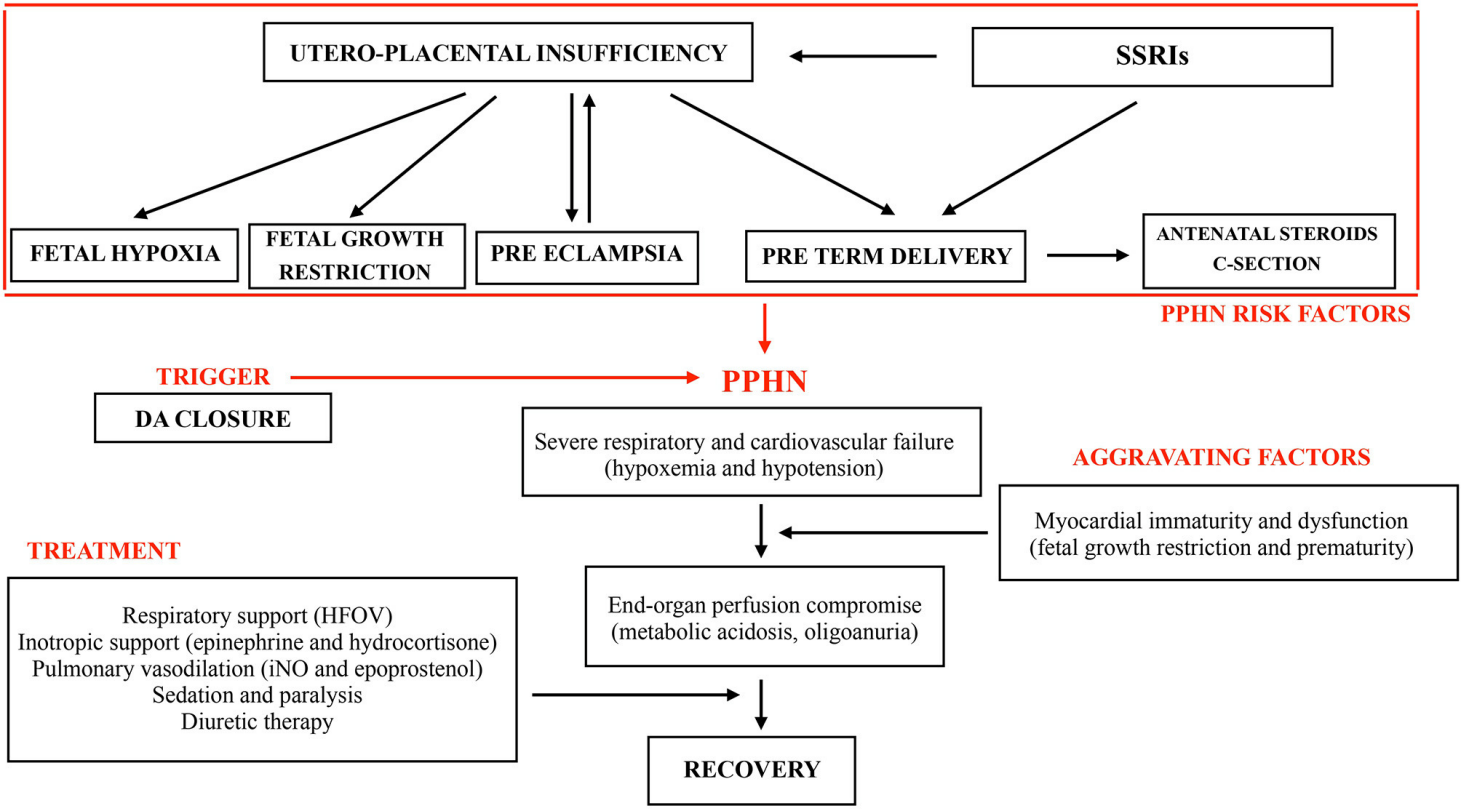
Physiopatological factors of PPHN, clinical course and interventions.

The clinical course of our patient and interventions are described in Figure 1.

The placenta has been described macroscopically according to the “Macroscopic Description Module of the Placenta” (10) and sampled in 14 paraffin blocks according to the 2016 Internal Instruction of the IRCCS Giannina Gaslini Institute. In particular, ten blocks represented the chorionic disc, with five of which documenting the maternal side with a strip of basal decidua.

**Figure 2:**
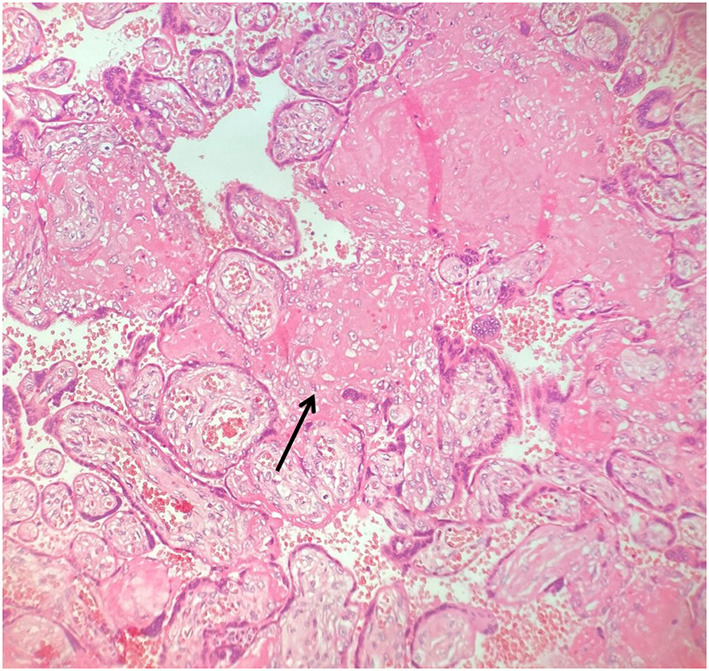
Placental villous changes (increased syncytial knots, villous agglutination, increased intervillous fibrin deposits - arrowhead).

The placental disc had a low weight (166 grams vs. 306 grams expected for the gestational age, <10° percentile) (11) with a birth weight-to-placental-weight ratio of 3.97. It presented changes associated with maternal malperfusion including villous changes (increased syncytial knots, villous agglutination) (Figure 2) and recent and intermediate small placental infarcts. The most characteristic aspect is chronic massive deposit of fibrinoid in subchorionic plate and at the peak of placental lobules without configuring massive transmural maternal floor infarct but reminding a maternal disreactive state of any etiology (12). The basal decidua did not show any lesion and/or vascular alteration. No signs of inflammation or infection has been highlighted in umbilical cord, membranes or chorionic plate.

No information about drug levels in maternal blood, amniotic fluid and cord blood are available.

## Discussion

PPHN is secondary to failure of pulmonary vascular transition at birth. The disorder is characterized by sustained elevation of PVR and extrapulmonary right-to-left shunting across persistent fetal channels (patent DA and FO). Shunting leads to severe hypoxemia and labile oxygen saturations. PPHN is most common among full-term or near-term infants, presenting shortly after birth with respiratory failure requiring various degree of intervention, from mechanical ventilation to extracorporeal membrane oxygenation (ECMO) support. Idiopathic PPHN in absence of significant lung disease is a rare finding in very low birth weight infants, in which hypoxemia typically results from surfactant deficiency, ventilation–perfusion mismatch and intrapulmonary shunting (4, 13). It is commonly believed that small premature newborns lack sufficient arteriolar vasculature to maintain prolonged elevated PVR after birth. However, in a retrospective study analyzing 15 PPHN premature newborns <30 weeks of GA weighting <1,500 g (14), the duration of ruptured membranes and use of antenatal steroids were significantly higher in the PPHN group. Excluding pPROM, not present in our patient, antenatal maternal steroids - as prevention of neonatal RDS - seem to accelerate pulmonary vascular smooth muscle development (14), acting as a potential risk factor for PPHN in preterm newborns. In addition, as discussed above, aside from extrapulmonary shunting, RDS -that is the typical respiratory feature of prematures- might generate a significant degree of intrapulmonary shunting and be associated with delayed postnatal circulatory adaptation characterized by pulmonary hypertension and prolonged ductal patency.

Recognized perinatal risk factors of PPHN reflect three physiopathological mechanisms: maladaptation, maldevelopment and underdevelopment (15). Maladaptation of the normally developed pulmonary vasculature results from an imbalance of vasoactive substrates: vasodilators, such as prostacyclin or endothelium-derived nitric oxide and vasoconstrictors, such as endothelin 1 and thromboxane A. Maladaptation is responsible for the greater part of PPHN cases and originates from sepsis, pneumonia, RDS, meconium aspiration, and fetal asphyxia. Maldevelopment of the pulmonary vasculature with excessive muscularization (increased smooth muscle cell thickness and distal extension of muscle to vessels) is mainly idiopathic, but sometimes is associated with chronic fetal hypoxia, fetal anemia or premature intrauterine closure of DA. Underdevelopment of pulmonary vasculature occurs in pulmonary hypoplasia, secondary to congenital diaphragmatic hernia or fetal renal disorders associated with oligohydramnios (7, 16). A number of other antenatal and perinatal risk-factors are reported, such as maternal smoking and asthma, causing fetal hypoxemia (17, 18), salicylate or other non-steroidal anti-inflammatory drugs, causing constriction of DA (19), and cesarean delivery (20).

SSRIs are widely prescribed to treat major depressive disorders (3). Untreated gestational depression may have adverse effects on the developing fetus (hyperactivity, irregular fetal heart rate), newborns (hormonal alterations, increased rates of premature deaths and neonatal intensive care unit admission), and children. On the contrary, the relationship between gestational depression and increased risks of prematurity and low birth weight remains controversial (21). Given this background, when making clinical decisions, clinicians should weight the growing evidence suggesting the detrimental and prolonged effects in offspring of untreated antenatal depression and depressive symptoms during pregnancy against the known and emerging concerns associated with in utero exposure to antidepressants. Maternal exposure to SSRIs during gestation is a known risk factor for PPHN. The first study that hypothesized this correlation was published by Chambers et al. (22) in 1996 in the New England Journal of Medicine. The same author validated this hypothesis 10 years later in a large case-control study (23). Other studies have been published on this controversial topic (24–27), with the most recent meta-analysis presented in 2019 (28) showing that the risk of PPHN is significantly increased in case of maternal exposure to SSRIs in any trimester. All gestational ages have been included, mostly full-term or near-term newborns, but no conclusive information regarding the effect of SSRIs in preterms can be deducted from this set of studies. The association between prenatal SSRIs exposure and pregnancy-outcomes with correlation to placental-histopathology has been recently reported in 82 cases of maternal SSRIs use matched with 82 controls (29). SSRI placentas were characterized by lower birth-weight-to-placental-weight ratio (6.0 ± 1.7) and higher rates of vascular maternal and fetal malperfusion lesions, as well-described in our case, where birthweight-to-placental-weight ratio was extremely low (3.97) and disreactive changes were significant. Even if we cannot relate these findings only to SSRI exposure, but also to maternal preeclampsia (30, 31), it is not possible to discriminate the weight of each factor. It is important to highlight that in this case significant changes typical of pre-eclampsia were not present (i.e., lesions and vascular alterations of the basal decidua). The SSRI group had lower birth weights, higher rates of NICU admission, mechanical ventilation and composite adverse neonatal outcome. Maternal and fetal vascular malperfusion lesions have been associated with preterm deliveries and fetal growth restriction (32). Of note, it is demonstrated that SSRIs increase the risk for preterm birth, with a biological mechanism not completely clear (3, 33). We considered the chronic exposure to SSRIs as the risk factor for fetal and placental changes and not the blood levels at time of delivery. According to the literature it is acceptable to measure neonatal outcomes based on dosage and duration of maternal exposure (3), assuming a correlation between maternal serum concentrations and concentrations in the fetal circulation (34) from data extrapolated in studies on citalopram pharmacokinetics.

The pathophysiological association between maternal use of SSRIs during pregnancy and PPHN remains not entirely understood. However, relevant findings are available. The lung acts as a reservoir for antidepressant drugs and substantial accumulation of SSRIs in the lungs has been reported (35, 36). SSRIs have direct and indirect vasoconstrictive properties: serotonin is itself a vasoconstrictive agent and has mitogenic effects on pulmonary smooth-muscle cells (37–39). SSRIs can inhibit the synthesis of NO, a vasodilator that regulates vascular tone and reactivity both in utero and during postnatal life (40, 41). Thus, higher circulating levels of serotonin and its accumulation in the fetal lung, combined with the reduced synthesis of NO, might result in proliferation of smooth-muscle cells and in imbalance of vasoactive substrates, creating a mix of maldevelopment and maladaptation mechanisms that leads to PPHN. As the clinical significance of this association is not universally supported (20, 42–44) and not completely clarified, it may be suggested that the combination of other factors may be crucial to generate PPHN. FGR - as hallmark of utero-placental insufficiency and chronic fetal hypoxia - is associated with pulmonary vascular remodeling and PH (45). Pregnancy-induced hypertension and preeclampsia are also expression of utero-placental insufficiency. In our case FGR, preeclampsia and cesarean section can be identified as strong co-factors for PPHN.

All these considerations support the hypothesis that our patient, exposed to chronic intrauterine hypoxia and to the potential adverse effects of maternal SSRIs use, developed structural and functional alterations of the pulmonary vascular system, setting up the physiopathological basis for severe PPHN. Risk factors and clinical course of our patient are described in Figure 1. The sudden onset characterized by desaturation, hemodynamic failure with severe metabolic acidosis and oliguria occurred with DA closure. We cannot exclude, as previously observed by Danhaive (46) in a series of premature infants, that the short time gap between ductal closure and the onset of PH, may suggest a cause - effect relationship between these two events. After birth, when PVR is still high but not greater than systemic, a left to right shunt through the DA may significantly increase the pulmonary blood flow and contribute to oxygenation and pulmonary vasodilation. The DA may constitute a significant pressure relief to the RV afterload when pulmonary arterial pressure is increased. Its physiologic or pharmacologic closure may trigger pulmonary vasoconstriction and impair pulmonary and systemic output, causing hypoxemia and hypotension, as occurred in our patient. Moreover, cyclooxygenase inhibitors and paracetamol not only decrease the synthesis of prostaglandin E2, a major mediator of ductal patency, but also of prostacyclin, a potent pulmonary vasodilator. Therefore, a cautious approach to DA closure should be mandatory, especially when PVR is still high in transitional circulation and risk factors for severe PH are present or suspected. In this case we did not consider to re-open the ductus to avoid the risk of pulmonary hemorrhage while maximal PH therapy was ongoing.

The extremely severe PPHN developed in our patient, refractory to maximal ventilatory and inotropic support and iNO, may be also the expression of the peculiar hemodynamic setting of this FGR preterm infant. The immature myocardium has limited capacity to adjust to the hemodynamic challenges in the immediate postnatal period due to fewer contractile elements, higher water content, greater surface-to-volume ratio and reliance on L-type calcium channels that utilize extracellular calcium. Moreover, fetal growth restriction is associated with myocardial remodeling and dysfunction (47). The association of these factors made this patient very vulnerable to significant circulatory compromise.

Focusing on SSRIs maternal exposure allowed us to study the physiopathology of PH in preterms. The most important limitation of this work is the presence of multiple potential factors involved in the clinical course of this preterm newborn, making difficult to identify the most relevant. In addition, placental histology is not specific but highly suggestive for SSRIs exposure, and this evidence was supported by previous studies.

In conclusion, maternal use of SSRIs during pregnancy, utero-placental insufficiency, FGR and prematurity leaded to severe PPHN, a life-threatening condition that requires complex supports and treatments. It has been stated that since untreated perinatal depression presents important adverse maternal and fetal outcomes, and the association between maternal SSRIs and PPHN remains of unclear significance, the potential damage caused by antidepressant drugs is likely outweighed by the benefit of such therapy (44). After reviewing the current evidence regarding the effects of SSRIs on placental pathology, risk of uteroplacental insufficiency, prematurity and severe pulmonary disease in the newborn, we would suggest to carefully evaluate the risk-benefit ratio of SSRIs use in selected cases of maternal depression. Moreover, when signs of preeclampsia are identified SSRI therapy should be re-considered for possible reduction and discontinuation. In addition, maternal SSRI therapy in pregnancy should be always identified when managing prematures for the high risk of severe PH with early ductal closure.

Further studies are required to advance current understanding and provide high quality recommendations in this setting.

## Data Availability Statement

The raw data supporting the conclusions of this article will be made available by the authors, without undue reservation.

## Ethics Statement

Ethical review and approval was not required for the study on human participants in accordance with the local legislation and institutional requirements. Written informed consent to participate in this study was provided by the participants' legal guardian/next of kin.

## Author Contributions

IB and SB conceptualized and designed the study, drafted the initial manuscript, and reviewed and revised the manuscript. MM, SP, EL, and FB designed the data collection instruments, collected data, carried out the initial analyses, and reviewed and revised the manuscript. EF and AM conceptualized and designed the study, coordinated and supervised data collection, and critically reviewed and revised the manuscript for important intellectual content. All authors contributed to manuscript revision, read, and approved the submitted version.

## Conflict of Interest

The authors declare that the research was conducted in the absence of any commercial or financial relationships that could be construed as a potential conflict of interest.

## Publisher's Note

All claims expressed in this article are solely those of the authors and do not necessarily represent those of their affiliated organizations, or those of the publisher, the editors and the reviewers. Any product that may be evaluated in this article, or claim that may be made by its manufacturer, is not guaranteed or endorsed by the publisher.

## Abbreviations

DA: Ductus Arteriosus
DOL: Day Of Life
FGR: Fetal Growth-Restricted or Fetal Growth Restriction
HFOV: High Frequency Oscillatory Ventilation
iNO: inhaled Nitric Oxide
PH: Pulmonary Hypertension
PPHN: Persistent Pulmonary Hypertension of the Newborn
PVR: Pulmonary Vascular Resistance
RDS: Respiratory Distress Syndrome
RV: Right Ventricle
SSRIs: Selective Serotonin Reuptake Inhibitors.
